# Expression of HER2 and Mismatch Repair Proteins in Surgically Resected Gallbladder Adenocarcinoma

**DOI:** 10.3389/fonc.2021.658564

**Published:** 2021-07-22

**Authors:** You-Na Sung, Sung Joo Kim, Sun-Young Jun, Changhoon Yoo, Kyu-Pyo Kim, Jae Hoon Lee, Dae Wook Hwang, Shin Hwang, Sang Soo Lee, Seung-Mo Hong

**Affiliations:** ^1^ Department of Pathology, Asan Medical Center, University of Ulsan College of Medicine, Seoul, South Korea; ^2^ Department of Pathology, Incheon St. Mary’s Hospital, College of Medicine, The Catholic University of Korea, Seoul, South Korea; ^3^ Department of Oncology, Asan Medical Center, University of Ulsan College of Medicine, Seoul, South Korea; ^4^ Department of Surgery, Asan Medical Center, University of Ulsan College of Medicine, Seoul, South Korea; ^5^ Department of Gastroenterology, Asan Medical Center, University of Ulsan College of Medicine, Seoul, South Korea

**Keywords:** gallbladder, cancer, mismatch repair proteins, microsatellite Instability, HER2

## Abstract

**Background:**

Gallbladder cancer (GBC) has a poor prognosis. Although complete surgical resection is the only successful approach for improving survival, additional therapeutic modalities are required for recurrent or surgically unresectable GBCs.

**Materials and Methods:**

To determine the expression status of HER2 and the mismatch repair (MMR) proteins MLH1, MSH2, MSH6, and PMS2, immunohistochemical staining of MMR proteins and HER2 was carried out in 216 surgically resected GBCs. HER2 labeling was scored by adopting a scoring system for gastric carcinomas. Tissues scoring 0 to 2+ were defined as HER2 negative, whereas those scoring 3+ were regarded as HER2-positive. In addition, silver *in situ* hybridization and microsatellite instability (MSI) analysis were conducted to confirm *HER2* amplification and MSI, respectively.

**Results:**

Three of 216 GBCs (1.3%) showed MMR protein deficiency. All three observed MSI cases exhibited dual loss of MSH2 and MSH6 protein expression. However, no cases showed loss of either MLH1 or PMS2 expression. No association was observed between MMR protein deficiency and other clinicopathological factors. *HER2* amplification was noted in 30 (13.9%) GBCs and associated with Crohn-like lymphoid reaction (P = 0.023). No survival difference was observed based on HER2 overexpression or *HER2* amplification status.

**Conclusion:**

MMR protein deficiency and HER2 overexpression were observed in a small subset (1.3% and 13.9%, respectively) of GBCs without simultaneous occurrence of deficient MMR protein expression and HER2 overexpression. The presence of Crohn-like lymphoid reaction may help identify cases with *HER2* amplification, by using hematoxylin-stained slides. Although the proportion of MMR protein-deficient- and HER2-overexpressing GBCs was small, applying immunotherapy to MMR protein-deficient GBCs and herceptin to HER2-overexpressing GBCs may provide alternative treatment options for patients with GBC.

## Introduction

Gallbladder cancer (GBC) is the most common malignancy of the biliary tract and is characterized by dismal prognosis (1, 2). The prevalence of GBC is relatively high in several countries, including Korea, Chile, Poland, India, Pakistan, Ecuador, and Japan, whereas being low in many Western countries (3). GBC and cholangiocarcinoma, is the seventh most common cancer in Korea (4). Currently, surgical resection is the only promising therapeutic option for cure. The median survival time of patients with GBC is 75 months, with a 5-year survival rate of about 50% after surgical resection (5). However, surgical resection is applicable only to up to 25% of all GBC patients exhibiting localized disease (6, 7). Conversely, systemic chemotherapies are required for patients with recurrent or metastatic GBC. Although gemcitabine and cisplatin are currently being used as the backbone of chemotherapeutic regimens for patients with GBC, the median overall survival for GBC patients treated with gemcitabine and cisplatin is less than 1 year (8, 9). Therefore, to improve the treatment response rate, a better understanding of the molecular mechanisms of GBC is pivotal.

Microsatellite instability (MSI), caused by abrogation of functional mismatch repair mechanisms, alters tumor biology by causing the inactivation of genes containing repeat sequences. For the cure of MSI-high metastatic colon cancer, the use of immune checkpoint inhibitors, such as pembrolizumab, was approved by the Food and Drug Administration (FDA) (10, 11). Subsequently, the FDA approvals for checkpoint inhibitors were extended to the treatment of all MSI-high solid tumors. Human epidermal growth factor 2 (HER2), a member of the receptor tyrosine kinase family, promotes cell proliferation thorough various signaling pathways (12). Therefore, blocking these HER2-dependent pathways has been considered a promising therapeutic strategy for patients harboring HER2-overexpressing or -amplified cancers; for instance, trastuzumab has been approved for the management of HER2-overexpressing breast and stomach cancers (13, 14). In addition, targeted exome sequencing of gallbladder cancer was recently introduced in clinical oncology for identifying druggable targets (15). Therefore, patients with gallbladder cancers with either MSI-high or HER2-overexpression or amplification could be used specific target agent. To date, several studies have examined the prevalence of HER2 overexpression (15–24) and DNA mismatch repair deficiency (25–31) in GBC. However, marked variations exist among these studies because of their relatively small sample size, varying experimental methods, and differing inclusion criteria. Therefore, to better understand the molecular biology and pathogenesis of GBC, systemic data analysis with a sufficient sample size is necessary.

The aim of this study was to investigate the expression status of MMR proteins and HER2 in patients with GBC, to provide a rationale for the application of immunotherapy and HER2-targeted therapy to GBC cases with advanced or metastatic setting.

## Materials And Methods

### Case Selection

This study was performed after approval from the institutional review board (approval number: 2019-0142) with a waiver of informed consent. A total of 216 surgically resected primary GBC patients were selected from a surgical pathology database. Clinical data, including patient’s age, sex, type of surgical resection, tumor location, survival time, and survival status, were reviewed in electronic medical records. Patients who had received neoadjuvant radiation and/or chemotherapy were excluded.

### Histopathologic Evaluation

Pathologic features, including tumor size, differentiation, histologic subtype, Crohn-like lymphoid reaction, and lymphovascular and perineural invasion, were reviewed by two pathologists (SMH and YNS). Histologic subtypes were classified according to the fifth edition of the World Health Organization (WHO) classification (32). T- and N-categories and stage grouping were evaluated according to the eighth American Joint Committee on Cancer (AJCC) staging system (33). Crohn-like lymphoid reaction was defined as discrete lymphoid aggregates with occasional germinal centers at the advanced edge of the tumor (34).

### Tissue Microarray Construction

Tissue microarrays (TMAs) were constructed from all 216 formalin-fixed, paraffin-embedded GBC tissue blocks by using a manual tissue micro-arrayer (Uni TMA Co, Ltd, Seoul, Korea). Areas occupied for >75% by tumor cells characterized by major histological differentiation and without accompanying tumor necrosis were selected, and four representative tumor tissue cores and one core from a matched normal gallbladder mucosal tissue were punched out from a donor block and transferred into a new recipient block with a punch of a 2-mm diameter.

### Immunohistochemical Staining of HER2 and MMR Proteins

Immunohistochemical (IHC) labeling was performed at the IHC laboratory of the Department of Pathology. Briefly, 4-μm-thick tissue sections from TMAs and whole-section slides of cases showing MMR protein deficiency in TMAs were deparaffinized and hydrated in xylene and serially diluted alcohol solutions. Endogenous peroxidase was blocked by incubation in 3% H_2_O_2_ for 10 min; next, heat-induced antigen retrieval was performed. Primary antibodies with BenchMark auto-stainers (Ventana Medical Systems, Tucson, AZ, USA) were used following the manufacturer’s protocol. Primary antibodies for HER2 (4B5, mouse monoclonal, 1:8, Ventana), MLH1 (clone M1, mouse monoclonal, prediluted, Roche, Basel, Switzerland), MSH2 (clone G219-1129, mouse monoclonal, 1:1000, Cell Marque, Rocklin, CA, USA), MSH6 (clone 44, mouse monoclonal, 1:200, Cell Marque), and PMS2 (clone EP51, rabbit monoclonal, 1:100, Dako, Glostrup, Denmark) were used. After the evaluation of IHC-labeled TMA slides, additional immunolabeling was performed on sections on the whole-section slides if samples showed any loss of MMR proteins or an HER2 immunolabeling score of 2+ or 3+ on TMA slides.

### HER2 IHC Scoring

**Figure 1:**
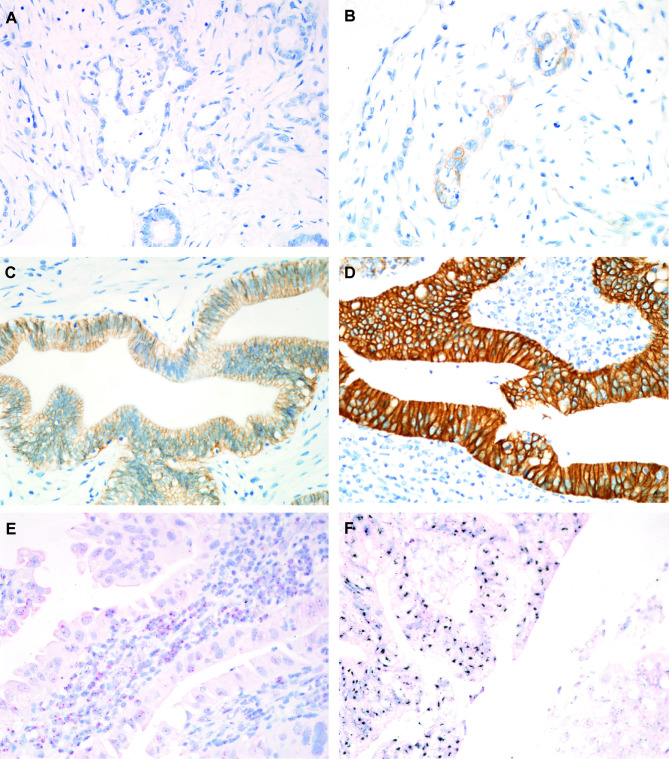
Representative images of HER2 immunohistochemical (IHC) staining and HER2 silver *in situ* hybridization (SISH). **(A)** HER2 IHC score 0, no expression (magnification, 400×); **(B)** HER2 IHC score 1+, barely visible (400×); **(C)** HER2 IHC score 2+, weak to moderate staining (400×); **(D)** HER2 IHC score 3+, intense staining (400×); **(E)** Absence of *HER2* amplification in cases with HER2 IHC score 0; **(F)** *HER2* amplification in cases with HER2 IHC score 3+. Red dots correspond to CEP17, and black dots correspond to HER2 (magnification, 400×).

HER2 IHC-sections were assigned a score from 0 to 3+ based on the intensity and pattern of membranous labeling, according to the scoring system recommended by the consensus panel on HER2 scoring for gastric cancer (35). Briefly, a HER2 IHC score of 0 (negative) was assigned for no or <10% reactivity in tumor tissue at 40× magnification; the score 1+ (negative) for faint/slight or partial (≥10%) membrane reactivity in tumor tissue at 40× magnification; the score 2+ (equivocal) for weak to moderate, complete or basolateral (≥10%) reactivity in tumor tissue at 10× to 20× magnification; and the score 3+ (positive) for moderate to strong, complete or basolateral (≥10%) reactivity in tumor tissue at 5× magnification [15]. Gastric cancer tissue with strong HER2 positivity (3+), confirmed to be effective in anti HER2 therapy, was used a positive control. Negative controls were performed by substituting a nonimmune purified mouse monoclonal antibody for the primary HER2 antibody. Tissues scoring from 0 to 2+ were defined as HER2 negative, whereas those with a score of 3+ were regarded as HER2 positive (**Figure 1**).

### HER2 Silver *In Situ* Hybridization and Scoring Criteria

Silver *in situ* hybridization (SISH) was conducted to confirm *HER2* amplification in all cases with a HER2 immunolabeling score of 2+ or 3+. Unstained slides were processed with an automated system following the manufacturer’s protocols to hybridize *HER2* and chromosome 17 (CEP17) probes (Ventana) (36). Both *HER2* and CEP17 probes were sequentially hybridized on the same slide. The *HER2* gene was visualized as a black dot and CEP17 as a red dot. The specimen was then counterstained with Harris hematoxylin.

The *HER2* amplification status was evaluated by counting *HER2* and CEP17 signals in nuclei of 20 consecutive tumor cells, according to the American Society of Clinical Oncology/College of American Pathologists (ASCO/CAP) guidelines (37). In brief, specimens were defined negative for *HER2* amplification if they displayed an *HER2*/CEP17 ratio of <2 and an average *HER2* copy number of <4.0 signals/cell; equivocal if they exhibited an *HER2*/CEP17 ratio of <2 and an average *HER2* copy number of ≥4.0 and <6.0 signals/cell; and positive if they showed an *HER2*/CEP17 ratio of ≥2 (37). Areas with overlapping nuclei, high nonspecific background staining, or weak signal were excluded from the evaluation (**Figure 1**).

### Analysis of MMR Protein Immunolabeling

Samples showing complete loss of nuclear staining for each MMR protein were defined as deficient for MMR proteins. Adjacent stromal cells and inflammatory cells with intact nuclear staining served as positive controls. For cases displaying loss of any MMR protein on TMA slides, MMR IHC staining was repeated on whole sections for histopathologic confirmation.

### MSI Analysis

Only in cases showing deficiency for MMR proteins on whole-sectioned slides, additional PCR was done to confirm the suspected MSI. Five quasi-monomorphic mononucleotide repeats, namely BAT25, BAT26, NR21, NR25, and NR27, were amplified in a single multiplex PCR reaction (38, 39). The PCR products were analyzed by capillary electrophoresis with an ABI310 Genetic Analyzer (Applied Biosystems, Foster City, CA, USA). Samples with MSI at ≥2 mononucleotide loci were considered MSI high, those with instability at a single mononucleotide locus MSI low, and those with no instability at any of the loci tested microsatellite stable (MSS), in accordance with National Cancer Institute (NCI) guidelines (40).

### Statistical Analysis

The R software (version 4.0.2, Vienna, Austria) was used to perform statistical analyses. The unpaired Student’s *t*-test was used to compare the means. Associations between HER2 expression and MSI status or other clinicopathologic factors were tested using the χ^2^ and/or Fisher exact tests. The overall survival rate was calculated using the Kaplan–Meier method, and significance was evaluated with the log-rank test. P<0.05 was considered to denote statistically significant differences.

## Results

### Clinicopathological Characteristics

The clinicopathological characteristics of the patients are summarized in **Table 1**. The mean age of the patients was 62.8 ± 10.1 years (range, 36–89 years) with a male-to-female ratio of 0.8. The mean tumor size was 3.4 ± 1.8 cm. One hundred and fifty-three cases (70.8%) were of biliary histologic subtype tumors, 10 cases (4.6%) were of intestinal subtype tumors, and 53 cases (24.6%) were of a mixed subtype. Ninety-seven (44.9%) cases consisted of moderately differentiated tumors. Based on the eighth edition of the AJCC cancer staging scheme, three carcinomas were *in situ* (Tis, 1.5%), 18 T1a (8.3%), 21 T1b (9.7%), 76 T2a (35.2%), 45 T2b (20.8%), and 53 T3 (24.6%). However, no T4 tumor was observed because all samples were obtained from surgically resected GBCs. Among the 127 evaluated cases with at least one examined lymph node, 70 were N0 (55.1%), 48 N1 (37.8%), and nine N2 (7.1%) tumors, according to the N category. Crohn-like lymphoid reaction was observed in 54 (25.0%) cases. Lymphovascular and perineural invasion was identified in 59 (27.3%) and 67 (31.0%) cases, respectively. 

**Table 1:** Clinicopathologic characteristics of the selected cases.

Characteristics		No. of patients	% of patients
**Sex**	Male	93	43.1
	Female	123	56.9
**Age**	<60	79	36.6
	≥60	137	63.4
**Size (cm)**	<3	92	42.6
	≥3	124	57.4
**Differentiation**	Well	81	37.5
	Moderate	97	44.9
	Poor	38	17.6
**Histologic subtype**	Biliary	153	70.8
	Intestinal	10	4.6
	Mixed	53	24.6
**T category (AJCC 8^th^)**	Tis	3	1.4
	T1a	18	8.3
	T1b	21	9.7
	T2a	76	35.2
	T2b	45	20.8
	T3	53	24.6
**N category (AJCC 8^th^)**	N0	70	55.1
	N1	48	37.8
	N2	9	7.1
**Lymphoid proliferation**	Absent	162	75.0
	Present	54	25.0
**Lymphovascular invasion**	Absent	157	72.7
	Present	59	27.3
**Perineural invasion**	Absent	149	69.0
	Present	67	31.0

### HER2 Immunolabeling and *HER2* SISH

**Figure 2:**
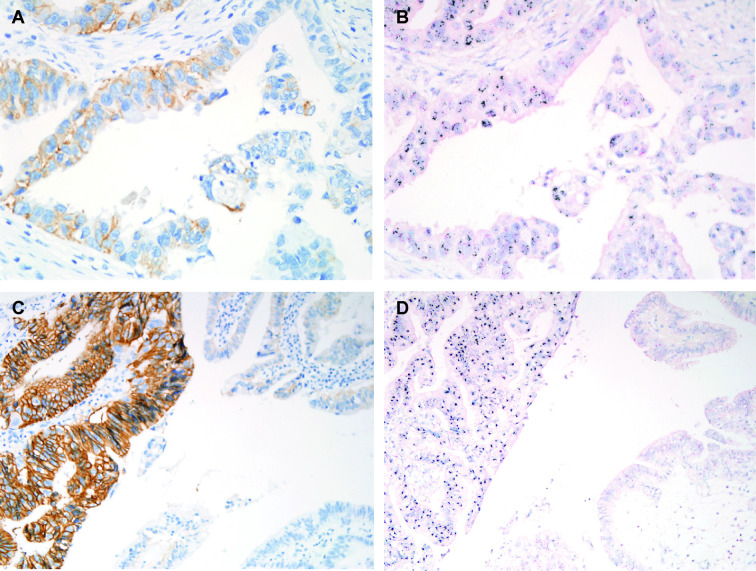
Representative images of matched HER2 immunohistochemical (IHC) staining and HER2 silver *in situ* hybridization (SISH) from the same case. **(A, B)** Weak to moderate HER2 IHC staining (**A**, left panel, 400×) corresponding to *HER2* amplification as shown by silver *in situ* hybridization (SISH; **B**, left panel, 400×). **(C, D)** Intense HER2 IHC staining at low magnification (**C**, left panel, 400×) corresponding to *HER2* amplification as shown by SISH (**D**, left panel, 400×). Red dots correspond to CEP17, and black dots correspond to HER2.

An IHC score of 0, 1+, 2+, and 3+ was observed in 172 (79.6%), 14 (6.5%), 14 (6.5%), and 16 (7.4%) cases, respectively (**Table 2**). Thirty GBC cases with IHC scores of 2+ or 3+ were evaluated by *HER2* SISH, and all exhibited *HER2* amplification. All cases with IHC score of 0 and 1+ showed no HER2 amplification (**Figure 1E**). Diffuse HER2 immunolabeling was noted in 67% (14 of 21) of HER2 IHC 2+ cases, whereas focal HER2 immunolabeling, characterized by a strong intensity of HER2 overexpression in association with HER2-negative areas, denoting heterogeneous HER2 expression, was observed in 33% (seven of 21) of GBC cases with an HER IHC score of 2+ on TMA slides (**Figure 2**). Similarly, diffuse HER2 immunolabeling was observed in 56% (9 of 16) of HER2 IHC 3+ cases; in contrast, 44% (seven of 16) of HER2 IHC 3+ tissues showed heterogeneous HER2 IHC labeling (focal HER2 IHC 3+). The results of correlation analysis between *HER2* amplification and other clinicopathologic factors are summarized in **Table 3**. Notably, there was no significant association between *HER2* amplification and clinicopathologic factors in patients with GBC.

**Table 2:** HER2 immunohistochemical staining score.

HER2 IHC score	No. of patients	% of patients
0	172	79.6
1+	14	6.5
2+	14	6.5
3+	16	7.4

**Table 3:** *HER2* amplification and microsatellite status in association with clinicopathologic characteristics in gallbladder cancer.

Characteristics	Variables	Amplified *HER2*	Unamplified *HER2*	P-value	Unstable microsatellite	Stable microsatellite	P-value
**Sex**	male	11 (36.7%)	82 (44.1%)	0.573	2 (66.7%)	91 (42.7%)	0.807
	female	19 (63.3%)	104 (55.9%)		1(33.3%)	122 (57.3%)	
**Age**	<60	13 (43.4%)	66 (35.5%)	0.533	1 (33.3%)	78 (36.6%)	1.000
	≥60	17 (56.7%)	120 (64.5%)		2 (66.7%)	135 (63.4%)	
**Size (cm)**	<3	11 (36.7%)	81 (43.5%)	0.611	0 (0%)	92 (43.2%)	0.360
	≥3	19 (63.3%)	105 (56.5%)		3 (100%)	121 (56.8%)	
**Differentiation**	Well	11 (36.7%)	70 (37.6%)	0.932	0 (0%)	81 (38.0%)	0.155
	Moderate	13 (43.3%)	84 (45.2%)		3 (100%)	94 (44.1%)	
	Poor	6 (20.0%)	32 (17.2%)		0 (0%)	38 (17.8%)	
**Histologic subtype**	Biliary	20 (66.7%)	133 (71.5%)	0.247	2 (66.7%)	151 (70.9%)	0.885
	Intestinal	0 (0%)	10 (5.4%)		0 (0%)	10 (4.7%)	
	Mixed	10 (33.3%)	43 (23.1%)		1(33.3%)	52 (24.4%)	
**T category**	Tis	0 (0%)	3 (1.6%)	0.350	0 (0%)	3 (1.4%)	0.633
	T1a	0 (0%)	18 (9.7%)		0 (0%)	18 (8.5%)	
	T1b	4 (13.3%)	17 (9.1%)		1 (33.3%)	20 (9.4%)	
	T2a	11(36.7%)	65 (34.9%)		0 (0%)	76 (35.7%)	
	T2b	9 (30%)	36 (19.4%)		1 (33.3%)	44 (20.7%)	
	T3	6 (20%)	47 (25.3%)		1 (33.3%)	52 (24.4%)	
**N category**	N0	11 (57.9%)	59 (54.6%)	0.729	1 (100%)	69 (54.8%)	0.663
	N1	6 (31.6%)	42 (38.9%)		0 (0%)	48 (38.1%)	
	N2	2 (10.5%)	7 (6.5%)		0 (0%)	9 (7.1%)	
**Lymphoid proliferation**	Absent	17 (56.7%)	145 (78.0%)	0.023*	1 (33.3%)	161 (75.6%)	0.314
	Present	13 (43.3%)	41 (22.0%)		2 (66.7%)	52 (24.4%)	
**Lymphovascular invasion**	Absent	20 (66.7%)	137 (73.7%)	0.564	3 (100%)	154 (72.3%)	0.677
	Present	10 (33.3%)	49 (26.3%)		0 (0%)	59 (27.7%)	
**Perineural invasion**	Absent	17 (56.7%)	132 (71.0%)	0.174	3 (100%)	146 (68.5%)	0.588
	Present	13 (43.3%)	54 (29.0%)		0 (0%)	67 (31.5%)	

*Significant at P < 0.05.

### Correlation Between HER2 Protein Expression, *HER2* Gene Amplification, and Clinical Outcomes

**Figure 3:**
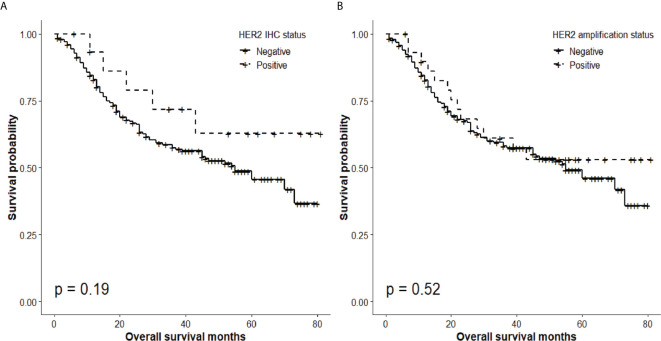
Overall survival according to HER2 status. **(A)** Absence of significant differences in overall 5-year survival rate according to HER2 IHC status (HER2 IHC 3+ tumors, 62.8%; HER2-negative (0, 1, 2+) tumors, 45.6%; P = 0.19). **(B)** Absence of significant differences in 5-year survival rate according to *HER2* amplification status (HER2 SISH+, 53.1%; HER2 SISH−, 45.9%; P = 0.52).

The overall 5-year survival rate of GBC patients expressing HER2 (IHC score, 3) was 62.8%, whereas that of patients not expressing HER2 (IHC score, 0 to 2) was 45.6%. Nevertheless, there was no significant difference in survival of GBC patients according to the HER expression status (P = 0.19; **Figure 3A**).

Similarly, although the overall 5-year survival rate of GBC patients with *HER2* gene amplification was 53.1%, whereas that of patients without *HER2* amplification was 45.9%, there was no significant difference in survival of patients with different *HER2* amplification status (P = 0.52; **Figure 3B**).

### MSI of GBC

**Figure 4:**
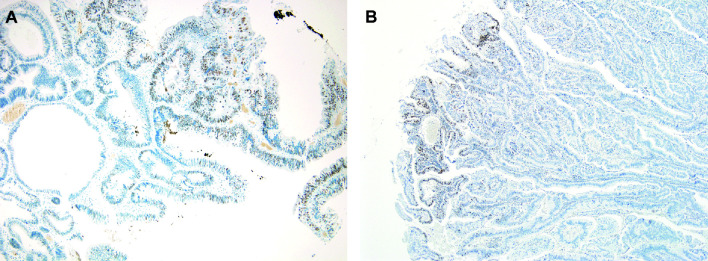
Heterogeneity of MMR protein immunolabeling. Loss of **(A)** MSH2 protein (left panel, 100×), and **(B)** MSH6 protein (right panel, 100×) with intact MMR proteins in another area of the same slide.

Fourteen (6.5%) out of the 216 analyzed GBCs showed loss of at least one MMR protein on TMA slides. Validation of MMR IHC on sections on the whole-section slides confirmed six of these 14 cases. Conversely, heterogeneous MMR protein expression was observed in the sections on the whole-section slides of the other eight cases (**Figure 4**). Finally, three (1.3%) GBC cases were confirmed as microsatellite unstable (two MSI high and one MSI low), and exhibited dual loss of MSH2 and MSH6. Furthermore, three cases displaying only MLH1 loss (case 6) or MSH6 loss (cases 11 and 14) in whole sections were MSS according to MSI PCR.

### Association Between HER2 and MMR Protein Expression Status and Other Clinicopathologic Factors

**Figure 5:**
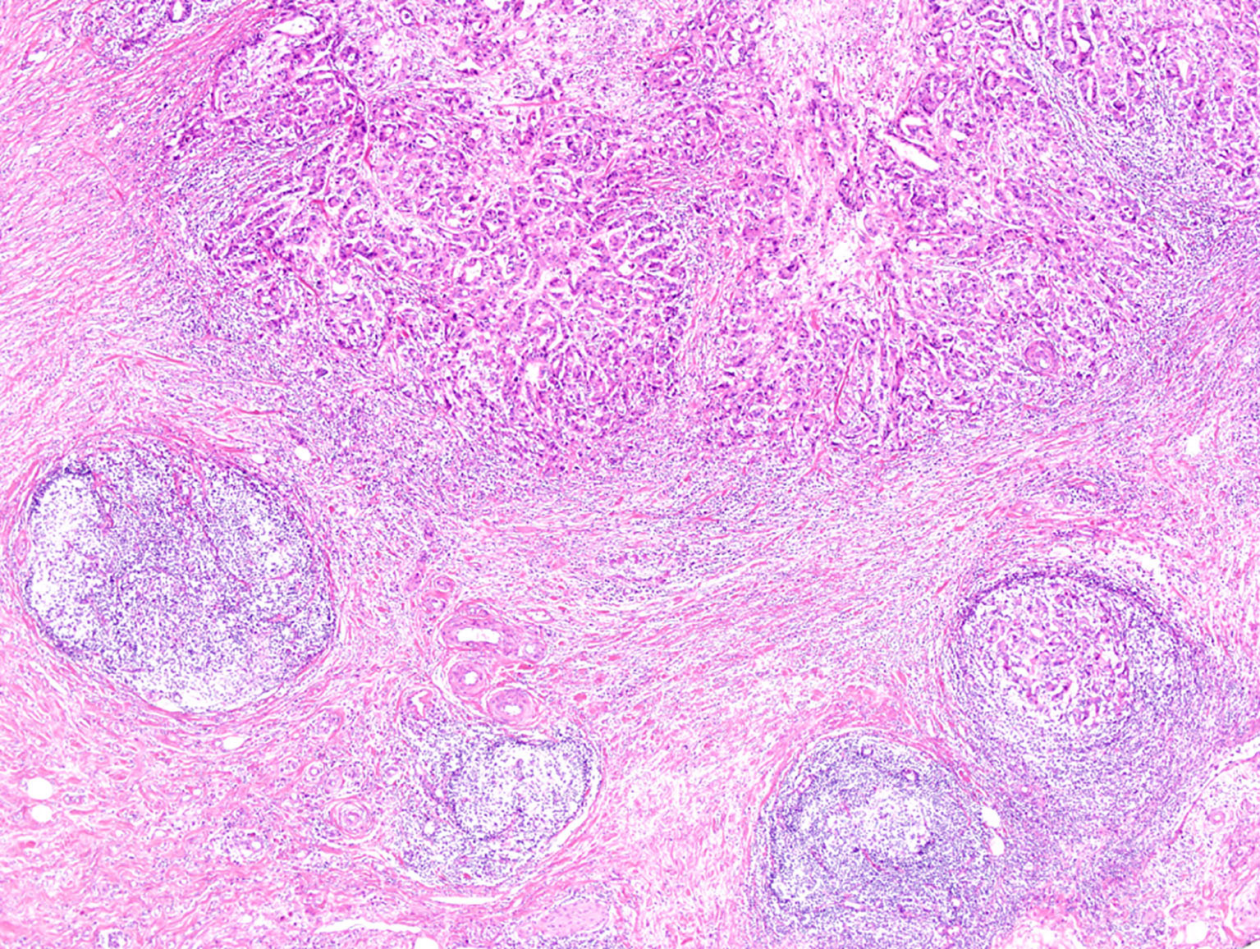
Representative image of Crohn-like lymphoid reaction observed at an advanced tumor edge (magnification, 40×).

Cases with *HER2* amplification were associated with Crohn-like lymphoid reaction (P = 0.023, **Figure 5**). On the other hand, although all three MSI-H GBC cases showed Crohn-like lymphoid reaction, there was no statistically significant association between these two factors because of the small number of MSI cases (three cases). In addition, there was no other association between MSI or *HER2* amplification status and other clinicopathologic factors (**Table 3**).

## Discussion

The present study, conducted in a large cohort, showed that about 14% of patients with GBC exhibited *HER2* amplification, and only 1% of them had high MSI. In particular, *HER2* amplification was observed in all the 30 cases that displayed an HER2 IHC score of 2+ or 3+.

The frequency of *HER2* amplification in the Korean cohort examined in the present study was about 14%. However, several previous studies with diverse ethnic backgrounds reported various frequencies of HER2 expression or *HER2* amplification in GBCs (**Table 4**) (16). In particular, the frequency of HER2 expression (IHC score >2+) in the previous studies ranged from 9.4% to 44% (16–21, 23, 24). Nevertheless, because most studies evaluated less than 100 GBC cases and applied different criteria for evaluating HER2 IHC results, direct comparison of the prevalence of HER2 expression was difficult. Only one recent study performed by Yoshida et al. (23) investigated HER2 expression within a large cohort (211 GBC patients) with the same HER2 IHC scoring system that we used in the present study; these authors found a frequency of HER2 ICH 2+ and 3+ GBCs of 6.6% (14/211) and 11.8% (25/211), respectively. Although the frequency of HER2 IHC 2+ tumors in the study by Yoshida et al. was similar to that of our study (6.5%), the proportion of GBCs with an HER2 IHC score of 3+ was slightly higher than that of the present study (7.4%). In addition, when investigating *HER2* amplification by *HER2* FISH, these authors found a quite different result from that of our study. Indeed, in our study, *HER2* amplification was observed in all tested cases scoring 2+ and 3+ for HER2 IHC. In contrast, a few GBC cases showed no *HER2* amplification (HER2 IHC score 2+: 2/12; IHC score 3+: 1/25) in the study by Yoshida et al. (23). Although both SISH and FISH are good methods to determine the amplification status, SISH is believed to be a better analytic method than FISH, because observation in bright fields enables more precise diagnosis, and lower bleaching risk allows longer-term storage of SISH samples (35). This could explain the difference in the observed amplification rate between the study of Yoshida et al. and the present study. Additionally, we observed an association between *HER2* amplification and Crohn-like lymphoid reaction. Similarly, tertiary lymphoid structures were more frequently observed in HER2-positive breast cancers (41). Moreover, an association between Crohn-like lymphoid reaction and *HER2* amplification status has been reported in urothelial carcinoma (42), and is probably related to HER2-dependent activation of inflammatory pathways, especially those connected with IL-1α and IL-6 (43, 44).

**Table 4:** Summary of previous studies on HER2 expression in gallbladder cancer.

Year	1^st^ author	Cohort	Case #	Frequency	Clinical significance	Method
2001	Kim	Korean	71	1+: 64%, 2+: 27%, 3+: 9%	Correlation with poor survival	Percentage of stained cells (1+: 5–33%, 2+: 34–66%, 3+: >67%)
2004	Matsuyama	Japanese	43	1+: 2%, 2+: 5%, 3+: 5%	No correlation with tumor grade	Intensity of membrane staining
2006	Chaube	Indian	78	+: 25%	HER2+ cases decreased with increasing grade, and age	Cytoplasm/membrane staining (+: >10%, −: <10%)
2007	Puhalla	Austrian	53	+: 13%	Correlation with advanced T stage	Complete or incomplete membrane staining (+/−)
2012	Kumari	Indian	97	2+: 4%, 3+: 9%	Gallstones present in 10 of 14 cases (3+ staining in seven cases and 2+ staining in three cases)	3+: Complete membrane staining >10% cells 2+: Incomplete membrane staining
2013	Roa	Chilean	187	0: 48.1%, 1+: 19%, 2+: 20%, 3+: 13%	More frequent in advanced/more differentiated cancers; worse survival in HER2+ patients	CAP/ASCO guidelines
2016	Yoshida	Japanese	211	0: 68%, 1+: 13%, 2+: 6.6%, 3+:12% Positive IHC and FISH: 17%	No correlation with clinicopathological factors or survival	IHC score of 3+ as ‘Modified IHC score in gastric cancer’ or 2+ with FISH positive result
2016	Pujani	Indian	25	0: 36%, 1+: 20%, 2+: 20%, 3+: 24%	No correlation with tumor stage, grade, and lymph node metastasis	CAP/ASCO guidelines

In the present study, we observed heterogeneous HER2 immunolabeling in 33% of HER2 IHC 2+ and 44% of HER2 IHC 3+ GBCs, which showed focal HER2 immunolabeling with locally strong HER2 labeling within a HER2-negative area. This result suggests that IHC examination of HER2 in samples from patients with GBC, by small endoscopic ultrasound-guided fine needle aspiration biopsy (EUS-FNAB) could lead to false-negative results, especially for tumors with unresectable or metastatic setting. Thus, to prevent possible HER2 IHC false negative results, it is required to get a larger number of cancer cells from metastatic or unresectable GBC lesions.

Most of the previous studies on HER2 expression included only IHC experiments (16–21, 24). In contrast, our present study confirmed the HER2 IHC score of 2+ in 14 cases and *HER2* amplification in all 14 cases by SISH.

MSI testing could be considered as a diagnostic approach for patients with GBC, because patients with MSI-high GBC stand a chance to benefit from immune checkpoint inhibitors (45). However, the proportion of MSI-high GBCs was found to be only 1.3% in the present study. The prevalence of various MSI conditions and their association with other clinicopathologic factors in GBC reported by previous studies are summarized in **Table 5**. In these studies, the prevalence of MSI in GBC varied widely from 0% to 26.5%, and no significant association with any clinicopathologic factor was identified. Although a few studies reported the association between MSI status and LOH or p53 mutation (25, 29), the number of cases was too small to draw a solid conclusion. We evaluated a relatively large number of surgically resected GBCs and observed only a small subset of MSI-high GBC cases (1.3%), with no obvious clinicopathologic association. Several previous papers evaluated PD-1 expression in prognostic aspect of gallbladder cancers (46–51), and one of the study reported association of MSI-H and PD-L1 expression (49). For precise evaluation of tumor infiltrating lymphocytes (TILs) and PD-L1 expression, TIL and PD-L1 expression should be evaluated mainly at the tumor-normal interphase, whole-section slides are required. However, the limitation of performing additional PD-L1 expression is that our present study is a TMA-based study. Therefore, further studies with large number of cases with whole sectioned slides are required for proper evaluation of MSI-H and association with PD-L1 expression.

**Table 5:** Summary of previous studies on microsatellite instability in gallbladder cancer.

Year	1^st^ author	Cohort	Frequency	Clinical/molecular significance	Method
2000	Yoshida	Japanese	5/30 (17%)	Inverse correlation between MSI and the presence of LOH	PCR (D2S123, D3S1029, D5S107, D17S261, D18S34)
2003	Sessa	Italian	0/71 (0%)	No association	PCR (BAT26) and IHC (MLH1 and MSH2)
2005	Roa	Chilean	6/59 (10%)	No association with clinicopathological variables or survival	PCR (BAT25, BAT26, D2S123, D5S346, and D17S250) and IHC
2006	Saetta	Greek	6/37 (16%)	No association with clinicopathological variables or survival	PCR (BAT25, BAT26, BAT40, D2S123, D5S346, and D17S250)
2008	Nagahashi	Japanese, Hungarian	9/34 (27%)	Associated with TP53 mutation	PCR (BAT-25, BAT-26, D2S123, D5S346, and D17S250)
2015	Moy	American	6/77 (8%)	No association with clinicopathological factors or survival	IHC (MSH2, MSH6, MLH1, and PMS2)
2009	Mishra	Indian	0/40 (0%)	No association	PCR

We observed heterogeneity of MMR protein expression, leading to a high false-negative rate in the evaluation of MSI status of GBCs, during the validation of IHC results of sections on the whole-section slides by PCR. Similar to the consequences of heterogeneous HER2 IHC staining, false-negative or false-positive results could be obtained during the evaluation of MMR protein expression or MSI status by EUS-FNAB on small samples from patients with GBC, especially for tumors with unresectable or metastatic setting.

The current findings indicate that subsets of patients with GBC may benefit from HER2-targeted therapy and anti-PD-1 therapy. Multiple anti-HER2 agents, including trastuzumab, HER2-based drug combinations, and HER2-directed antibody-drug conjugates, are now under investigation for treating patients with HER2-overexpressing biliary tract cancer, based on their promising efficacy in prior small retrospective studies (52). As pembrolizumab, an anti-PD-1 agent, was recently approved for the management of patients harboring MSI-high tumors, regardless of cancer type (53–55), a greater effort should be made to identify MSI-high GBC patients, considering the limited therapeutic options and the poor prognosis of recurring or metastatic GBC cases.

In summary, MMR protein deficiency and HER2 overexpression were observed in a small subset (1.3% and 13.9%, respectively) of GBCs. There was no simultaneous occurrence of deficient MMR protein expression and HER2 overexpression. The occurrence of Crohn-like lymphoid reaction could help identify GBC cases with *HER2* amplification on hematoxylin-stained slides. Although the proportion of MMR protein-deficient and HER2-overexpressing tumors was small, applying immunotherapy to MMR protein-deficient GBCs and trastuzumab to HER2-overexpressing GBCs may provide additional treatment options for patients with surgically resected GBC.

## Data Availability Statement

The raw data supporting the conclusions of this article will be made available by the authors, without undue reservation.

## Ethics Statement

The studies involving human participants were reviewed and approved by Institutional Review Board of Asan Medical Center (approval number: 2019-0142). Written informed consent for participation was not required for this study in accordance with the national legislation and the institutional requirements.

## Author Contributions

Conceptualization, Y-NS and S-MH. Methodology, Y-NS and S-MH. Software, Y-NS. Investigation, Y-NS, S-YJ, and S-MH. Data curation and formal analysis, SK. JL, DH, SL, and S-MH. Resources, DH, SH, and S-MH. Writing—original draft preparation, Y-NS, S-YJ. Writing—review and editing, Y-NS, CY, K-PK, and S-MH. Visualization, Y-NS. Supervision, S-MH. Project administration, Y-NS. All authors contributed to the article and approved the submitted version.

## Conflict of Interest

The authors declare that the research was conducted in the absence of any commercial or financial relationships that could be construed as a potential conflict of interest.
